# Accurate prediction of functional effect of single amino acid variants with deep learning

**DOI:** 10.1016/j.csbj.2023.11.017

**Published:** 2023-11-10

**Authors:** Houssemeddine Derbel, Zhongming Zhao, Qian Liu

**Affiliations:** a Nevada Institute of Personalized Medicine, University of Nevada, Las Vegas, Las Vegas, NV 89154, USA; b Center for Precision Health, McWilliams School of Biomedical Informatics, The University of Texas Health Science Center at Houston, Houston, TX 77030, USA; c School of Life Sciences, College of Sciences, University of Nevada, Las Vegas, Las Vegas, NV 89154, USA

**Keywords:** Functional effect, Deep learning, Single amino acid variant, Precise estimation, High-throughput experiments

## Abstract

The assessment of functional effect of amino acid variants is a critical biological problem in proteomics for clinical medicine and protein engineering. Although natively occurring variants offer insights into deleterious variants, high-throughput deep mutational experiments enable comprehensive investigation of amino acid variants for a given protein. However, these mutational experiments are too expensive to dissect millions of variants on thousands of proteins. Thus, computational approaches have been proposed, but they heavily rely on hand-crafted evolutionary conservation, limiting their accuracy. Recent advancement in transformers provides a promising solution to precisely estimate the functional effects of protein variants on high-throughput experimental data. Here, we introduce a novel deep learning model, namely Rep2Mut-V2, which leverages learned representation from transformer models. Rep2Mut-V2 significantly enhances the prediction accuracy for 27 types of measurements of functional effects of protein variants. In the evaluation of 38 protein datasets with 118,933 single amino acid variants, Rep2Mut-V2 achieved an average Spearman’s correlation coefficient of 0.7. This surpasses the performance of six state-of-the-art methods, including the recently released methods ESM, DeepSequence and EVE. Even with limited training data, Rep2Mut-V2 outperforms ESM and DeepSequence, showing its potential to extend high-throughput experimental analysis for more protein variants to reduce experimental cost. In conclusion, Rep2Mut-V2 provides accurate predictions of the functional effects of single amino acid variants of protein coding sequences. This tool can significantly aid in the interpretation of variants in human disease studies.

## Graphical Abstract

**Figure:**
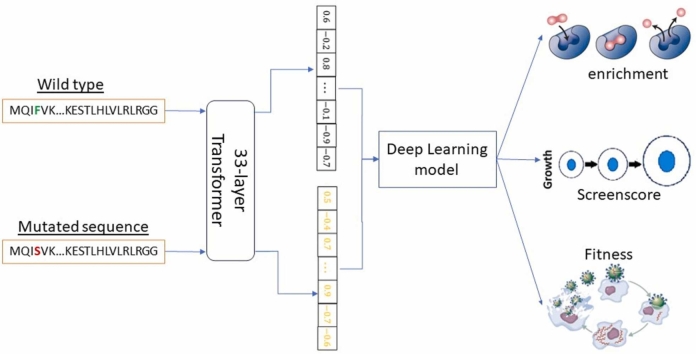

## 1 Introduction

Proteins play fundamental roles in carrying out diverse cellular functions. Their biological activities could be affected by numerous variants. Although most of these variants have negligible effects on protein functions, a small fraction of native amino acid variants of human proteins are closely associated with human diseases [1]. Additionally, synthetically introduced variants are crucial in protein engineering to design proteins with specific characteristics. In both applications, accurately estimating the functional effects of millions of protein variants is a fundamental and challenging problem.

High-throughput experimental approaches, deep mutational scanning[2] and the recently developed GigaAssay[3], are proposed to address this challenge [4], [5]. These methods introduce thousands of variants of a given protein and measure the variants’ functional effects on the biological activities of the protein, overcoming the limitation of interpreting limited, natively occurring variants of proteins. These high-throughput experimental approaches have successfully and comprehensively investigated the functional effects of various variants for tens of proteins, including *influenza Hemagglutinin*[6] *and influenza A virus PA polymerase subunit*[7]*, nonstructural protein 5A (NS5A) inhibitor for Hepatitis C virus*[8]*, Hsp90*[9]*, beta-lactamase TEM-1*[10], [11], [12], [13]*, a bacterial DNA methyltransferase M.HaeIII*[14]*, BRCA1*[15]*, hYAP65 WW domain*[16]*, a Tn5 transposon-derived kinase APH(3′)II*[17]*, yeast transcription factor Gal4*[18]*, human tumor suppressor p53*[18]*, PDZ domain*[19]*, Saccharomyces cerevisiae poly(A)-binding protein Pab1*[20]*, yeast ubiquitin gene*[21], [22], [23] *and a tRNA gene*[24]*, β-glucosidase enzyme Bgl3 from Streptomyces sp.*[25]*, FAS/CD95*[26]*, 20 ParD-ParE TA family members*[27]*, murine ubiquitination factor E4B*[28] *and HIV Tat*[3]. However, as indicated by the list, each experiment typically focused on one protein/gene due to the high cost of the methods. It is difficult, if not impossible, to extend these methods to examine variants of thousands of proteins or to investigate all possible multiple variants of a given protein.

Thus, computational approaches were proposed to overcome this limitation. These approaches can be classified into four groups. The first group, which includes PolyPhen-2[29], SIFT[30] and SNAP2[31], usually relies on evolutionary conservation of homologous sequences[32], while the second group, which includes CADD[33], integrates diverse annotations in the inference of mutational effects. The third group, which includes EVmutation[34] and DeepSequence[35], considers epistatic couplings between amino acids[36], [37]. DeepSequence also uses a variational auto-encoder to detect latent features in sequences to predict a variant’s functional effect. The fourth group of the methods, which includes transformer models such as Evolutionary Scale Modeling (ESM) [38], [39], has recently been used to automatically capture higher-order hidden information behind the sequences. The transformer models were trained on millions of available protein sequences, and provide a novel way to predict the mutational effects of protein variants without the need for hand-craft features. However, few studies investigated how learned features from transformers perform on those high-throughput experimental datasets with various mutational measurements of functional effects upon protein variants.

Here, we propose a deep learning method, named Rep2Mut-V2, to use protein sequences as the sole input to accurately predict 27 types of measurements of mutational effects of protein variants. Rep2Mut-V2 is an improvement of our previous model Rep2Mut [40] which was designed to predict the transcriptional activity of HIV Tat protein (GigaAssay [3]). In an assessment of 38 protein datasets, Rep2Mut-V2 demonstrated superior performance when compared to six existing methods. Rep2Mut-V2 exhibits great potential to assist the investigation of mutational effects for more proteins, aiding in the interpretation of protein variants and human disease studies. Our tool is publicly available at https://github.com/qgenlab/Rep2Mut.

## 2 Materials and methods

### 2.1 Datasets

A total of 38 protein datasets, comprising 118,933 single amino acid variants (Table 1), were used to evaluate our method and the state-of-the-art methods. Each of these datasets investigates a protein with various numbers of variants, and the number of variants range from 313 (on YAP1 dataset) to 12,236 (on BF520 dataset) with a median of 1725 as shown in Table 1. Each dataset is also associated with a specific functional measurement, such as transcriptional activities, fitness, CRIPT, MIC score, etc. [35]. The 38 datasets encompass a total of 27 distinct functional measurements. Most of the datasets were generated by deep mutational scanning and collected by Riesselman et al. [35], while the HIV Tat data was generated by GigaAssay[3].

**Table 1:** The 38 datasets for testing the model. Among them, fitness is used as the measurement for six datasets, and these six datasets are BF520, BG505, P84126, POLG_HCVJF, TIM_SULSO, and TIM_THEMA. “#variants”: the number of variants; “Seq length”: the length of protein sequences.

**Dataset names**	**Short Dataset names**	**#variants**	**Seq length**
AMIE_PSEAE_Whitehead	AMIE	4,507	346
B3VI55_LIPSTSTABLE	B3VI55_LIPSTSTABLE	6,541	439
B3VI55_LIPST_Whitehead2015	B3VI55_LIPST	6,327	439
BF520_env_Bloom2018	BF520	12,236	852
BG505_env_Bloom2018	BG505	12,217	860
BG_STRSQ_hmmerbit	BG_STRSQ	2,635	501
BLAT_ECOLX_Ostermeier2014	BLAT_2014	4,595	286
BLAT_ECOLX_Palzkill2012	BLAT_2012	4,789	286
BLAT_ECOLX_Ranganathan2015	BLAT_2015	4,788	286
BLAT_ECOLX_Tenaillon2013	BLAT_2013	949	286
BRCA1_HUMAN_BRCT	BRCA1_BRCT	1,185	1,863
BRCA1_HUMAN_RING	BRCA1_RING	492	1,863
CALM1_HUMAN_Roth2017	CALM1_Roth2017	1,730	149
DLG4_RAT_Ranganathan2012	DLG4_RAT	1,558	724
GAL4_YEAST_Shendure2015	GAL4	1,104	881
HG_FLU_Bloom2016	HG_FLU	10,337	565
HSP82_YEAST_Bolon2016	HSP82	4,104	709
IF1_ECOLI_Kishony	IF1_ECOLI	1,312	72
MK01_HUMAN_Johannessen	MK01	5,463	360
MTH3_HAEAESTABILIZED_Tawfik2015	MTH3	1,719	330
P84126_THETH_b0	P84126	1,519	254
PABP_YEAST_Fields2013-singles	PABP	1,187	577
PA_FLU_Sun2015	PA_FLU	1,848	716
POLG_HCVJF_Sun2014	POLG_HCVJF	1,631	3,033
PTEN_HUMAN_Fowler2018	PTEN	3,014	403
RASH_HUMAN_Kuriyan	RASH	3,078	189
RL401_YEAST_Bolon2013	RL401_2013	1,160	76
RL401_YEAST_Bolon2014	RL401_2014	1,294	76
RL401_YEAST_Fraser2016	RL401_2016	1,168	76
SUMO1_HUMAN_Roth2017	SUMO1	1,329	101
TIM_SULSO_b0	TIM_SULSO	1,519	248
TIM_THEMA_b0	TIM_THEMA	1,519	252
TPK1_HUMAN_Roth2017	TPK1_2017	2,608	243
TPMT_HUMAN_Fowler2018	TPMT_2018	2,659	245
UBC9_HUMAN_Roth2017	UBC9	2,281	159
UBE4B_MOUSE_Klevit2013-singles	UBE4B	603	1,173
YAP1_HUMAN_Fields2012-singles	YAP1	313	504
HIV_Tat	HIV_Tat	1,615	86

### 2.2 Deep learning framework to predict functional effects of protein variants

**Fig. 1:**
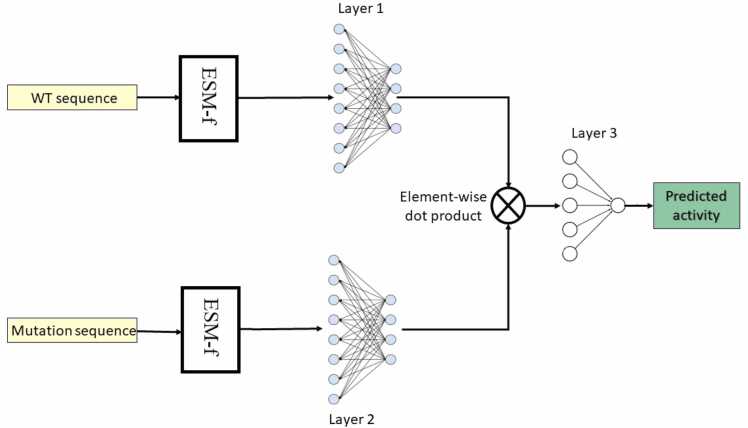
The architecture of Rep2Mut-V2 to predict mutational effect from protein sequences. The model consists of 328,067 trainable parameters. “ESM” is used to generate representation vectors only; therefore, we denote it as “ESM-f” to distinguish from ESM prediction.

Rep2Mut-V2 is a deep learning-based method to estimate various functional effects of protein variants. Rep2Mut-V2 uses a pair of protein sequences as input, i.e*.*, a wildtype sequence (WT), and a mutated sequence with a substitution of an amino acid at a position of interest (Fig. 1). Rep2Mut-V2 uses the WT and mutated sequences as input of ESM to learn the representation of the mutated position [38] (denoted as “ESM-f”, which distinguishes ESM predictions). ESM-f is composed of multiple transformer layers and is trained on millions of protein sequences with the masked language modeling objective. It endeavors to learn multiple levels of protein knowledge such as biochemical properties and evolutionary information. ESM-f has several different releases, and ESM-1v, used in Rep2Mut-V2, comprises 34 transformer layers trained on the UniRef90 dataset [41]. The 33rd layer of ESM-1v generates a 1,280-element vector which is used to represent either WT or variant information for the position of interest in the protein sequence. Each of the representation vectors is then used as the input of a fully connected neural network layer with a vector of 128 elements as output (Layers 1 and 2 in Fig. 1). After that, the two 128-dimension vectors are merged using an entry-wise product and then fed into Layer 3 to estimate the functional effect of a variant. The entry-wise product (or Hadamard product) takes two matrices of the same dimensions as inputs and generates another matrix of the same dimension as the operands using a binary operation. For example, given two matrices *A*_*m,n*_ and *B*_*m,n*_ with *m × n* dimensions, the entry-wise product $A⊙B={\left(A\right)}_{\mathrm{ij}}{\left(B\right)}_{\mathrm{ij}}$ where *0 <i ≤ m*, and *0 <j ≤ n*. Additionally, a PReLU activation function [42] and a dropout rate of 0.2 are applied to the fully connected neural network layers to avoid overfitting.

### 2.3 Training and testing Rep2Mut-V2

Three different strategies are used to evaluate Rep2Mut-V2: The first strategy is ten-fold cross-validation. With this strategy, Rep2Mut-V2 is assessed in two steps: a leave-one dataset-out pretraining step, and a cross-validation fine-tuning step. Initially, 36 of 37 non-GigaAssay datasets are used to pretrain the deep learning framework to capture shared information across proteins. During this step, layers 1 and 2 are shared across the datasets, and each dataset has its own specific layer 3. The framework was pretrained using 10 epochs with a batch size of 256 and a learning rate of 1e-5. After that, the remaining dataset is randomly split into 10 groups, and each group contains ∼10% variants. Each time, 90% of the variants of the dataset are used for fine-tuning, and the other 10% are for testing. The fine-tuning process is conducted with a batch size of 8, and a learning rate of 1e-4 for layer 3, and 5e-6 for layers 1 and 2. Both pretraining and fine-tuning processes use the Adam optimizer [43] and MSE loss function in the back-propagation process. *MSE* is defined in Eq. (1) where *n* is the number of variants, *Y*_*i*_ are the observed activities and *Ŷ* are the predicted activities.

(1) $$\mathrm{MSE}=\frac{1}{n}\;\sum_{i=1}^{n}{\left(Y_{i}-{\hat{Y}}_{i}\right)}^{2}$$

To compare Rep2Mut-V2 with other methods and to avoid the influence of random split of datasets, the fine-tuning process was repeated 50 times, and the final evaluation was based on the averaged performance.

The second strategy is few-shot learning. The few-shot learning model is trained on a small fraction of variants, but can be used to accurately predict the functional effects of a wide array of variants. Our few-shot learning process is evaluated on six datasets with fitness measurement (in Table 1), as other measurements are used on relatively fewer available datasets. During few-shot learning, we use 5 out of 6 fitness datasets for pretraining, as we did previously. Then, 30% of the variants of the remaining dataset are used for the fine-tuning, and the remaining 70% are used for testing. The testing is repeated five times on each of 6 fitness datasets.

The third strategy is zero-shot learning, where a new dataset that is not used in training process is used for testing. It allows the use of our model without further training. To evaluate zero-shot performance, we train the model using 5 out of 6 fitness datasets, and test our model on the 6th dataset. This process is repeated six times for each fitness dataset.

### 2.4 Estimate mutational effects of protein variants with state-of-the-art methods

We evaluated the performance of Rep2Mut-V2 against six published methods that were described below.

*ESM*: The first published method is ESM [38], [39], a pretrained model to estimate protein’s activity and functions. The ESM approach estimates the functional effects of protein variants through the following process: Given a protein sequence, ESM produces a representation vector of each position; Then, an additional layer is added to calculate a probability vector of all amino acid types for each position in the sequence [39]; after that, given a position of interest, the amino acid in the wildtype protein serves as a reference state and is compared to the mutated amino acid type. The variant effect is then calculated using the logarithmic ratio of the probability between the mutated amino acid and the WT amino acid [39] as shown below:

(2) $$\sum_{t\in T}\log p\left(x_{t}=x_{t}^{\mathrm{mt}}|,x_{\backslash T}\right)-\log p\left(x_{t}=x_{t}^{\mathrm{wt}}|,x_{\backslash T}\right)\;$$

where p is a probability vector for a position of interest, T is the set of mutated positions for a variant, $x_{\mathrm{\backslash T}}$ is the masked input sequence, $x_{t}^{\mathrm{mt}}$ and $x_{t}^{\mathrm{wt}}$ represent the mutant and wildtype amino acids, $p\left(x_{t}=x_{t}^{\mathrm{mt}}|,x_{\mathrm{\backslash T}}\right)$ is the probability assigned to the mutated amino acid $\;x_{t}^{\mathrm{mt}}$, and $p\left(x_{t}=x_{t}^{\mathrm{wt}}|,x_{\mathrm{\backslash T}}\right)$ is the probability assigned to the wildtype.

ESM released two versions (ESM-v1 and ESM-v2) each with several pretrained models. For ESM-v1, we employed two models in this evaluation: esm1v_t33_650M_UR90S_1 [39] (denoted as ESM-M2) if the protein sequence length is shorter or equal to 1024, and esm1_t34_670M_UR50S [38] (denoted as ESM-M1) for all datasets due to its ability to handle protein sequences longer than 1024 amino acids. ESM-2 [44] (denoted as ESM-v2) uses more parameters: 36 layers with up to 15 billion parameters vs 33 layers used in ESM-M1 and ESM-M2. The used ESM-v2 model is esm2_t36_3B_UR50D.

*DeepSequence*: DeepSequence [35] is a deep latent-variable model. It uses the concept of variational autoencoders (VAE) [45] to extract latent factors from a protein (or RNA) sequence, and can capture higher-order correlations in biological sequence families. Although DeepSequence is a generative model, predicting a variant’s effect on each protein sequence requires additional training. Given a sequence, we used a multiple sequence alignment (MSA) tool to generate MSA sequences. As recommended tools by DeepSequence, we used EVcoupling from the website v2.evcouplings.org together with a bit score of 0.5 bits/residue as a threshold during MSA. These MSA sequences were then used to retrain DeepSequence for predicting mutational effects.

*SIFT*: Sorting Intolerant From Tolerant (SIFT) [30] is an old tool to predict a variant’s effect on protein function. It uses substitution tolerance of a protein position to estimate the variant’s effect. Given a sequence, SIFT usually collects a set of related sequences and aligns them against the target protein. Then, it calculates the degree of conservation of amino acids and uses this to estimate a score that specifies whether a variant is tolerated or deleterious.

*CPT*: Cross-protein transfer (CPT) [46] uses various features to predict a variant’s effect. These features include scores from EVE and ESM-1v, MSAs, structural features from AlphaFold2, as well as amino acid descriptors such as charge, polarity, hydrophobicity, size, local flexibility, and so on. Based on these features, CPT uses a linear regression algorithm to train a model on five human proteins (CALM1, MTHR, SUMO1, UBC9, and TPK1). The model is mainly evaluated on human proteins for clinical variant interpretation. The variant’s predictions on human proteins were downloaded and used for performance evaluation.

*VariPred*: VariPred [47] is another ESM-based approach to predict pathogenicity of amino acid variants. Its manuscript was released on bioRxiv after our initial submission. It uses ESM to generate vector representations for predicting a variant’s pathogenicity. Its prediction outcome is binary: 1 denotes pathogenic and 0 means not pathogenic. We also extracted VariPred’s predictions for human proteins from our 38 datasets and evaluated its performance.

EVE: evolutionary model of variant effect (EVE) [48], like DeepSequence, utilizes evolutionary information to predict the clinical significance of human variants. It uses multiple sequence alignment (MSA) as input to train a Bayesian Variational autoencoder (VAE), and estimates an evolutionary index to distinguish variant sequences and wild-type sequences.

### 2.5 Evaluation measurements

We use the Spearman's correlation coefficient (SRCC) to measure the performance of each tested method across the 38 datasets. For the implementation, we used the Python package scipy to calculate the SRCC between the predicted and experimental estimates. Specifically, we let *X* and *Y* be experimental and predicted estimates for a list of variants. SRCC is calculated using Eq. (3).

(3) $$\mathrm{SRCC}=\frac{\mathrm{cov}(R\left(X\right),R(Y))}{\sigma_{R(X)}\sigma_{R(Y)}}$$

where $R\left(*\right)$ is the ranking of items in *, $\mathrm{cov}(R\left(X\right),R(Y))$ is the covariance of X and Y, $\sigma_{R(X)}$ is the standard deviation of X, and $\sigma_{R(Y)}$ is the standard deviation of Y.

## 3 Results

### 3.1 Evaluation of Rep2Mut-V2 under zero-shot and few shot strategies

Rep2Mut-V2 was first evaluated under three strategies: zero-shot transfer learning, few-shot learning and leave-one position-out cross-validation. The evaluation was conducted on the six fitness datasets (as detailed in Table 1), because other measurements were not available for more datasets and different measurements may not be comparable.

The zero-shot learning model was tested on a dataset after being trained on other fitness datasets. Through leave-one dataset-out cross-validation, the results in Table 2 reveal that Rep2Mut-V2 achieved better performance on three of the six datasets when compared with ESM, and on four of the six datasets when compared with DeepSequence.

**Table 2:** The performance on the six fitness datasets. The values are Spearman’s rank correlation coefficients.

	**One position out (no transfer)**	**Zero-shot transfer**	**few-shot transfer**	**DeepSequence**	**ESM-M1**	**ESM-M2**
BF520	0.632	0.735	**0.789**	0.003	0.37	0.469
BG505	0.627	0.738	**0.776**	0.179	0.37	0.481
P84126	0.564	0.45	**0.71**	0.588	0.569	0.536
POLG_HCVJF	0.61	0.440	**0.651**	0.372	0.069	
TIM_SULSO	0.557	0.495	**0.622**	0.551	0.608	0.595
TIM_THEMA	0.47	0.467	**0.608**	0.446	0.468	0.48

Few-shot learning usually fine-tunes a model on a small fraction of variants of a dataset and then tests the model on other variants of the dataset, after being pretrained on other datasets. In our evaluation, we pretrained our few-shot learning model on five of six datasets, and fine-tuned the model using 10% of the variants of the remaining testing dataset. The model was assessed on 90% of the variants of the testing dataset. The results are presented in Table 2 which shows that Rep2Mut-V2 outperforms DeepSequence and ESM on all six datasets.

We further assessed Rep2Mut-V2 using leave-one position-out cross-validation, because mutated positions might be correlated with fitness measurements. In our cross-validation, training and testing data were split based on mutated positions, rather than randomly splitting. As presented in Table 2, Rep2Mut-v2 still generates better results than state of the arts models on most of datasets. Rep2Mut-v2 outperformed DeepSequence, ESM-1 and ESM-2 by average SRCC improvements of 0.269, 0.265 and 0.112 respectively.

### 3.2 Evaluation of Rep2Mut-V2 with ten-fold cross-validation

**Fig. 2:**
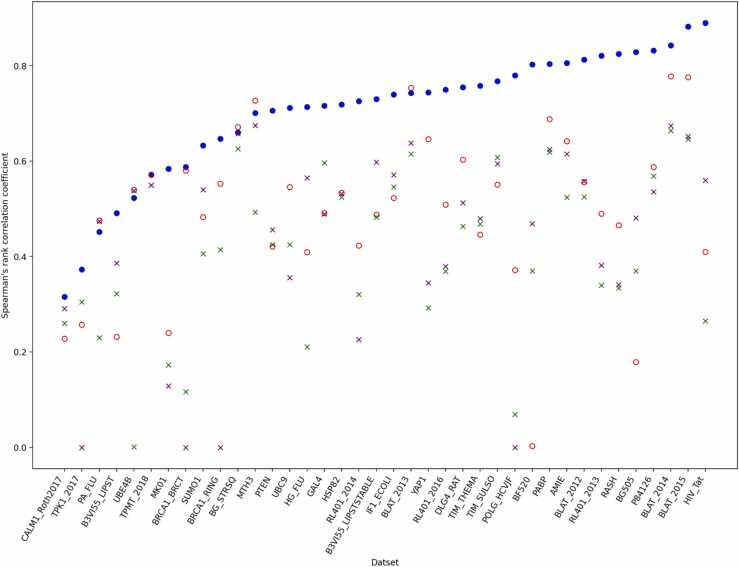
The performance (Spearman’s rank correlation) of Rep2Mut-V2 on different datasets compared to the two state-of-the-arts methods. Blue filled circles: Rep2Mut-V2, red empty circles: DeepSequence, Green crosses: ESM-M1, and purple crosses: ESM-M2.

The performance of Rep2Mut-V2 on the 38 datasets under ten-fold cross-validation is presented in Table 3 and Fig. 2, together with the predictions made by SIFT, ESM and DeepSequence on the 38 datasets. The results generated by CPT and VariPred on human proteins are also provided in Table 3, considering that the developers of CPT and VariPred mainly assessed the predictions of variants’ effect on human proteins.

**Table 3:** The performance (Spearman’s rank correlation) achieved by Rep2Mut-V2, DeepSequence, two ESM predictions (i.e., ESM-M1: esm1_t34_670M_UR50S, and ESM-M2: esm1v_t33_650M_UR90S_1), SIFT, CPT and VariPred. Na: Not available, because ESM-M2 cannot process a protein sequence longer than 1024 amino acids. DeepSequence's results are mainly borrowed from [35] except the last dataset, The results of CPT and EVE were downloaded from https://huggingface.co/spaces/songlab/CPT and https://evemodel.org/ respectively.

**Dataset names**	**Rep2Mut-V2**	**DeepSequence**	**ESM-M1**	**ESM-M2**	SIFT	CPT	VariPred	EVE	ESM-v2
AMIE	**0.806**	0.642	0.524	0.615	0.317				0.685
B3VI55_LIPSTSTABLE	**0.73**	0.488	0.483	0.598	0.257				0.632
B3VI55_LIPST	**0.491**	0.232	0.322	0.386	0.139				0.39
BF520	**0.803**	0.003	0.37	0.469	0.35				0.131
BG505	**0.829**	0.179	0.37	0.481	0.355				0.093
BG_STRSQ	0.66	**0.672**	0.626	0.658	0.506				0.649
BLAT_2014	**0.843**	0.778	0.664	0.674	0.563				0.611
BLAT_2012	**0.813**	0.556	0.525	0.558	0.458				0.53
BLAT_2015	**0.882**	0.776	0.646	0.652	0.488				0.589
BLAT_2013	0.743	**0.754**	0.615	0.638	0.494				0.587
BRCA1_BRCT	**0.588**	0.580	0.117		0.354	0.54	0.429	0.586	0.502
BRCA1_RING	**0.647**	0.553	0.414		0.399	0.55	0.466	0.567	0.505
CALM1_Roth2017	**0.316**	0.228	0.26	0.291	0.199	0.307	0.077	0.233	0.276
DLG4_RAT	**0.755**	0.603	0.463	0.513	0.313				0.46
GAL4	**0.716**	0.491	0.597	0.489	0.067				0.696
HG_FLU	**0.714**	0.409	0.211	0.565	0.357				0.558
HSP82	**0.719**	0.534	0.524	0.532	0.245				0.482
IF1_ECOLI	**0.74**	0.523	0.546	0.571	0.3				0.576
MK01	**0.584**	0.240	0.173	0.129	0.051	0.184	0.075		0.189
MTH3	0.701	**0.727**	0.493	0.675	0.247				0.608
P84126	**0.832**	0.588	0.569	0.536	0.262				0.595
PABP	**0.804**	0.688	0.619	0.625	0.278				0.628
PA_FLU	0.452	**0.476**	0.23	0.474	0.439				0.19
POLG_HCVJF	**0.78**	0.372	0.069		0.572				0.134
PTEN	**0.706**	0.421	0.425	0.456	0.251	0.54			0.317
RASH	**0.825**	0.466	0.334	0.341	0.21	0.456	0.182	0.477	0.439
RL401_2013	**0.821**	0.490	0.34	0.382	0.38				0.55
RL401_2014	**0.726**	0.423	0.321	0.226	0.431				0.432
RL401_2016	**0.75**	0.509	0.369	0.379	0.394				0.57
SUMO1	0.633	0.483	0.406	0.54	0.42	**0.641**	0.318		0.468
TIM_SULSO	**0.768**	0.551	0.608	0.595	0.329				0.632
TIM_THEMA	**0.758**	0.446	0.468	0.48	0.236				0.47
TPK1_2017	0.373	0.257	0.305		0.255	0.387	0.224	0.231	**0.403**
TPMT_2018	0.572	0.571	0.55	0.55	0.257	**0.593**		0.357	0.503
UBC9	**0.712**	0.546	0.425	0.356	0.432	0.585	0.316		0.556
UBE4B	0.523	**0.540**	0.001	0.538	0.334				0.347
YAP1	**0.744**	0.646	0.292	0.345	0.147	0.55	0.463	0.616	0.486
HIV_Tat	**0.89**	0.41	0.265	0.56	0.265				0.053

**Fig. 3:**
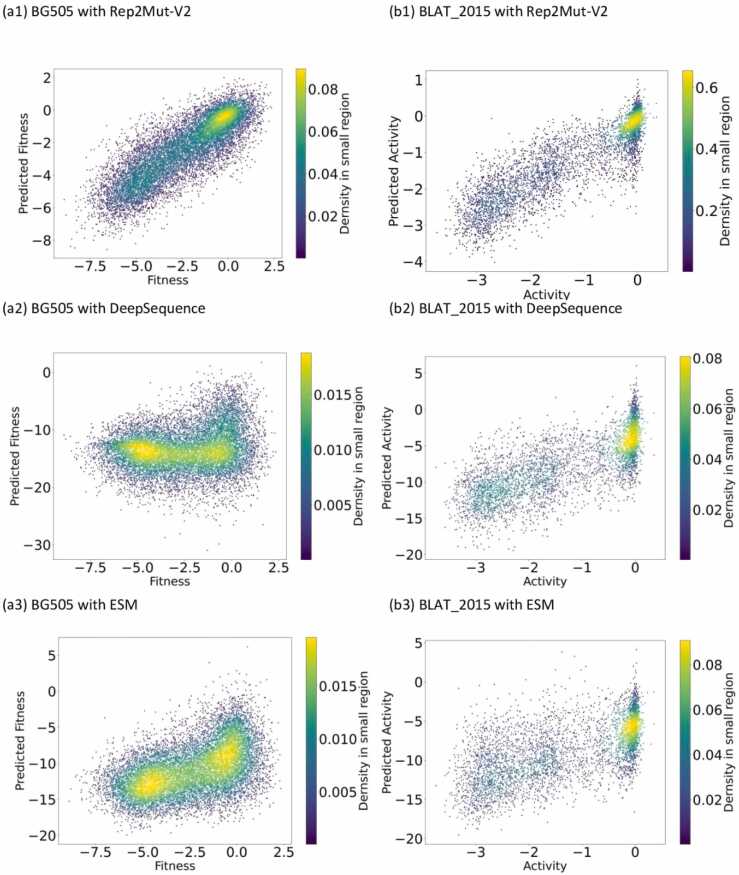
Comparison of the estimation of mutational effects by Rep2Mut and two state-of-the-art methods (DeepSequence and ESM). a: BG505 dataset, b: BLAT_2015. 1: Rep2Mut-V2, 2: DeepSequence, 3: ESM.

Fig. 2 illustrates the significant improvement of our Rep2Mut-V2 approach over DeepSequence and ESM. Rep2Mut-V2 outperformed DeepSequence on 33 datasets, while DeepSequence showed better performance on five datasets. The SRCC improvement by Rep2Mut-V2 ranges from 0.001 (on TPMT_2018) to 0.8 (on BF520), averaging 0.242. In contrast, the SRCC improvement by DeepSequence varied from 0.011 (on BLAT_2013) to 0.026 (on MTH3), averaging 0.018. Notably, our improvement is substantially higher than that achieved by DeepSequence. For instance, on BG505 dataset, Rep2Mut-V2 yielded a SRCC of 0.829, while DeepSequence’s SRCC is 0.179, as shown in Fig. 3(a1, a2). On this dataset, DeepSequence’s prediction is almost random compared to the experimental estimation, and Rep2Mut-V2’s prediction closely aligns with the experimental estimation with an average error of approximately 0.171. On another dataset, BLAT_2015, the SRCCs of Rep2Mut-V2 and DeepSequence were 0.882 and 0.776, respectively (Fig. 3(b1, b2)). Rep2Mut-V2 demonstrates a 0.106 improvement against DeepSequence in this dataset.

When compared against ESM, Rep2Mut-V2 notably outperformed ESM-M1 on all 38 datasets: the improvement in SRCC ranges from 0.034 (on BG_STRSQ) to 0.711 (on POLG_HCVJF), with an average SRCC improvement of 0.29. Furthermore, Rep2Mut-V2 demonstrated better performance than ESM-M2 on 36 datasets, with a median SRCC improvement of 0.209. The SRCC improvement ranged from 0.002 (on BG_STRSQ) to 0.50 (on RL401_2014). The datasets of PA_FLU and UBE4B were exceptions where ESM-M2 outperformed Rep2Mut-V2 by 0.022 and 0.015, respectively. But overall, Rep2Mut-V2’s predictions outshine ESM. For example, on the BG505 dataset in Fig. 3 (a1, a3), Rep2Mut-V2 achieved a SRCC of 0.829, while ESM-M2 scored 0.481. Rep2Mut-V2’s SRCC is 0.348 higher than ESM-M2. Similarly, on the BLAT_2015 dataset (Fig. 3 (b1, b3)), ESM-M2 (SRCC: 0.652) produced poorer performance than Rep2Mut-V2 (SRCC: 0.882). Recently, ESM released the 2rd version (ESM-V2), and we tested the performance of ESM-V2 to predict functional effects as shown in Table 3. ESM-v2 achieved similar results to ESM-M1, ESM-M2, and performed worse than Rep2Mut-V2.

When compared to SIFT and EVE, Rep2Mut-V2 exhibited a consistent trend of performance improvement, outperforming both SIFT and EVE on all tested datasets. The SRCC improvement over SIFT ranged from 0.013 (PA_FLU) to 0.649 (GAL4), with an average improvement of 0.378. The SRCC improvement over EVE ranged from 0.002 (BRCA1_BRCT) to 0.348 (RASH), averaging 0.142.

In the evaluation against VariPred, Rep2Mut-V2 excelled, surpassing VariPred on all 11 human datasets. The average improvement varied from 0.149 (TPK1_2017) to 0.643 (RASH), averaging 0.319. Similarly, Rep2Mut-v2 improved the prediction variants’ effects on 8 out of 11 human datasets when compared to CPT, with the SRCC improvement ranging from 0.009 (CALM1_Roth2017) to 0.4 (MK01).

In summary, the estimation of Rep2Mut-V2 is substantially superior to DeepSequence, ESM, SIFT, CPT, VariPred and EVE. The averaged SRCC of Rep2Mut-V2 is 0.703, while DeepSequence, ESM-M1, ESM-M2, ESM-V2, SIFT, CPT, VariPred, and EVE had averaged SRCC score of 0.496, 0.412, 0.496, 0.461, 0.325, 0.484, 0.283 and 0.438 respectively. Rep2Mut-V2 significantly outperforms the state-of-the-art methods.

### 3.3 Performance of Rep2Mut-V2 on smaller training data

The above assessment of Rep2Mut-V2 used 90% of the dataset for training and 10% for testing. However, it is generally expensive and time consuming to generate more variant data through wet-lab experiments. Therefore, we tested Rep2Mut-V2’s performance with less variants for training. In detail, we used 30% of the variants from a dataset for fine-tuning Rep2Mut-V2 and the remaining 70% variants for testing.

**Fig. 4:**
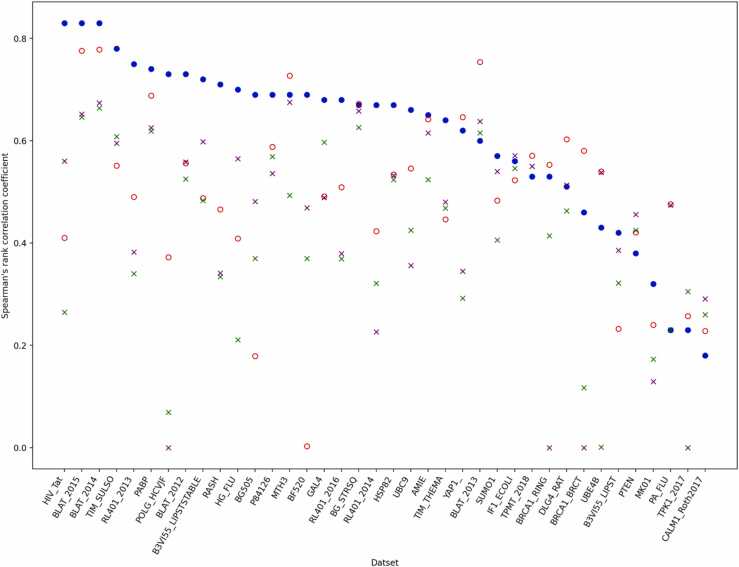
The performance (Spearman’s rank correlation) of Rep2Mut-V2 comparing to DeepSequence, ESM-M1 and ESM-M2. Rep2Mut-V2 was trained with less variants.

We compared this Rep2Mut-V2 model to DeepSequence and ESM. As illustrated in Fig. 4, this Rep2Mut-V2 model still outperformed DeepSequence ion 26 datasets and ESM on 29 datasets. Compared to Rep2Mut-V2 with 90% variants for training, the performance of this Rep2Mut-V2 model decreased by 0.077 points on average. This robust performance clearly demonstrates Rep2Mut-V2’s ability to generate accurate predictions for variant analysis, especially in cases with limited available variants determined by wet-lab experiments. This offers a better solution for analyzing functional effect for numerous variants to reduce the intensive financial and human resources required in wet-lab experiments.

## 4 Discussion

Rep2Mut-V2 was evaluated on 118,933 single amino acid variants from 38 protein datasets with 27 types of measurements of functional effects. The evaluation was conducted under various cross-validation strategies, and the performance of Rep2Mut-V2 was compared against six existing methods. The results consistently highlighted the superiority of Rep2Mut-V2 in accurately predicting the mutational effects upon protein variants. Even using limited variants for training, Rep2Mut-V2 maintained superior performance over existing methods. Notably, Rep2Mut-V2 relies solely on protein sequences and does not require protein 3D structures for precise prediction. Given the availability of millions of protein sequences compared to the limited number of proteins with experimental 3D structures, Rep2Mut-V2 proves to be a highly valuable tool, especially for those proteins which have experimentally determined functional effects only for a small fraction of variants.

It is important to note that Rep2Mut-V2 uses representation vectors generated from an ESM framework as input, but its prediction performance is higher than the prediction performance of both ESM and DeepSequence. This success is partially attributed to the fact that ESM prediction relies solely on representation vectors of WT sequences, whereas our method uses representation vectors of both WT sequences and mutant sequences as inputs. Our design allows the models to learn the difference between WT and mutant vectors for accurate prediction. On the other hand, DeepSequence depends on evolutionary data generated from multiple sequencing alignments to infer mutational effects. Its performance is thus limited by the availability of similar sequences to refine the model.

Our method has some limitations. First, our method was designed to predict the functional effects of single amino acid variants and is not currently equipped to handle high-order variants. We are presently extending our framework to address the prediction of functional effects of double/triple variants, although there are limited datasets with high-order variants. Second, we tested transfer learning with Rep2Mut-V2. However, transfer learning generally treats the contribution of each dataset equally, while the reliability of measuring functional effects of variants is different across those datasets. This uniform treatment may mislead transfer learning. To overcome this, a potential solution is to weigh the contribution of each dataset to shared layers (in Fig. 1) based on experimental reliability of the measurement of functional effects. Unfortunately, with only 38 experimental protein datasets, it is hard to arrive at a robust conclusion. As the functional effects of more protein variants are experimentally determined through high-throughput methods, transfer learning could effectively learn shared information across proteins and then substantially enhance the prediction of variants’ effects. Consequently, these two limitations will be overcome with the availability of more datasets.

## 5 Conclusion

In this study, we proposed and tested Rep2Mut-V2 across 38 protein datasets with various effect measurements. Our approach was compared with six existing methods, and the evaluation demonstrates that our approach can achieve much better performance on most of the datasets. By relying solely on protein sequences, our approach achieved accurate prediction of functional effects even with a limited number of variants for training. These observations strongly suggest that Rep2Mut-V2 has the potential to study mutational effects across a broader spectrum of proteins, thereby benefiting human disease studies.

## Declaration of Competing Interest

The authors claim that there is no conflict of interest.
